# Structure of a putative immature form of a Rieske-type iron-sulfur protein in complex with zinc chloride

**DOI:** 10.1038/s42004-023-01000-6

**Published:** 2023-09-09

**Authors:** Erika Tsutsumi, Satomi Niwa, Ryota Takeda, Natsuki Sakamoto, Kei Okatsu, Shuya Fukai, Hideo Ago, Satoshi Nagao, Hiroshi Sekiguchi, Kazuki Takeda

**Affiliations:** 1 Department of Chemistry, Graduate School of Science, Kyoto University, Sakyo-ku, Kyoto 606-8502 Japan; 2 RIKEN SPring-8 Center, 1-1-1 Kouto, Sayo-cho, Sayo-gun, Hyogo 679-5148 Japan; 3 Japan Synchrotron Radiation Research Institute, 1-1-1 Kouto, Sayo-cho, Sayo-gun, Hyogo 679-5198 Japan

**Keywords:** X-ray crystallography, Metalloproteins, Enzyme mechanisms

## Abstract

Iron-sulfur clusters are prosthetic groups of proteins involved in various biological processes. However, details of the immature state of the iron-sulfur cluster into proteins have not yet been elucidated. We report here the first structural analysis of the Zn-containing form of a Rieske-type iron-sulfur protein, PetA, from *Thermochromatium tepidum* (TtPetA) by X-ray crystallography and small-angle X-ray scattering analysis. The Zn-containing form of TtPetA was indicated to be a dimer in solution. The zinc ion adopts a regular tetra-coordination with two chloride ions and two cysteine residues. Only a histidine residue in the cluster-binding site exhibited a conformational difference from the [2Fe-2S] containing form. The Zn-containing structure indicates that the conformation of the cluster binding site is already constructed and stabilized before insertion of [2Fe-2S]. The binding mode of ZnCl_2_, similar to the [2Fe-2S] cluster, suggests that the zinc ions might be involved in the insertion of the [2Fe-2S] cluster.

## Abstract (web summary)

Iron–sulfur clusters are important cofactors involved in a wide range of protein functions, however, the incorporation of FeS clusters into proteins remains underexplored. Here, the authors report the crystal structure of a Zn-containing form of a Rieske protein, in which ZnCl_2_ binds in an FeS-binding site, representing a putative immature form of a Rieske protein prior to FeS insertion.

## Introduction

Iron-sulfur clusters are prosthetic groups involved as critical protein components in a wide range of protein functions, including respiration, photosynthesis, DNA repair, sensing of substances and various enzymatic reactions^1,2^. These various roles are thought to be evidence of the significant influences of iron-sulfur clusters on the development and evolution of life^3,4^. While various types of iron-sulfur clusters have been discovered as protein cofactors, the rhomboid [2Fe-2S] cluster and the cubane [4Fe-4S] cluster are the most widely observed type^3,5,6^.

Iron-sulfur clusters are synthesized in vivo by protein machineries such as iron sulfur cluster (ISC), sulfur mobilization (SUF) and nitrogen fixation systems consisting of various component proteins^7–9^. The systems have the essentially same synthesis mechanism and are composed of proteins with similar functions. In all systems, cysteine desulfurases catalyze the assembly step by binding the sulfur atom of cysteine to iron in order to assemble iron-sulfur clusters in scaffold proteins. The synthesized FeS clusters are transferred to receptor proteins in the insertion step regulated by molecular chaperones and carrier proteins. Insufficiency in iron-sulfur cluster biogenesis leads to various diseases in humans, such as Friedreich’s ataxia, Parkinson’s disease and some types of mitochondrial disease^10–14^.

The bacterial ISC system is similar to the eukaryotic ISC system and the central component proteins are homologous each other, while some differences exist between the two on details of the regulatory proteins and chaperone mechanisms^15–19^. This indicates the existence of a common ancestral system, and further suggests that the biogenesis of iron-sulfur clusters has remained important from the earliest stages of life. Therefore, it is expected that the essence of the function of more complicated eukaryotic systems can be extracted by examining simpler bacterial iron-sulfur cluster synthesis systems. In the bacterial ISC system, the assembly step is progressed by cysteine desulfurase IscS, scaffold protein IscU, iron donor IscX, electron donor ferredoxin and activity modulator frataxin, and inserted to target proteins by monothiol glutaredoxin GrxD and chaperones HscA/HscB. The structures of several ISC proteins and their complexes have been determined for both eukaryotic and bacterial systems^20–29^. In recent years, there has been progressed in the understanding of the assembly step for both bacterial and mammalian systems^30–33^, whereas the insertion step from the complex to the target protein is still poorly understood. Thus, the molecular details of the entire maturation process of iron-sulfur proteins are still unclear.

Although the eukaryotic cytochrome *bc*_1_ complex composed of 11 subunits^34,35^, the bacterial complex is composed of only three subunits^36,37^. Both complexes form homodimeric structures. Rieske protein PetA, one of the three subunits of the bacterial cytochrome *bc*_1_ complex, functions as an electron transfer pathway in the photosynthesis of photosynthetic bacteria as well as in the respiration of aerobic bacteria^38^. PetA has a hydrophilic globular domain containing one Rieske-type [2Fe-2S] cluster and a single transmembrane α-helix. The two portions are linked by a flexible hinge region. The globular domain is exposed on the periplasmic side of the plasma membrane^36,37^.

In this paper, we report structural analyses of the Zn-containing form of the PetA of a photosynthetic purple bacterium, *Thermochromatium tepidum* (TtPetA)^39^. Based on structural comparison between the Zn-containing form and the [2Fe-2S]-containing form, we discuss about a protection mechanism of the immature cluster binding site and further provide implication for the insertion process of the iron-sulfur cluster.

## Results

### Characterization of purified TtPetA

**Fig. 1:**
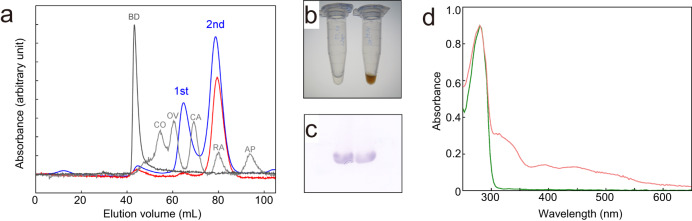
Characterization of TtPetA. **a** The elution profiles of the SEC monitored with absorbance at 280 nm and 380 nm are shown in blue and red, respectively. The profile of the marker mixture is shown in gray. CO conalbumin, OV ovalbumin, CA, carbonic anhydrase, RA ribonuclease A, AP aprotinin. The profile for blue dextran (BD) is shown in dark gray. **b** Photograph of the purified samples. The left tube is for the first peak of SEC, while the right tube is for the second peak. **c** Immunoblotting for the first and second peaks. The left lane is for the first peak, while the right lane is for the second peak. The photograph is flipped horizontally from left to right from the original version to match the order in (**a**) and (**b**). **d** UV–visible absorption spectra of the first (green) and second (red) peaks.

The soluble domain of TtPetA was heterologously expressed in *Escherichia coli*. TtPetA was divided into two peaks in the purification step with size-exclusion chromatography (SEC) (Fig. 1a). The first elution peak had a molecular mass of 41 kDa, which corresponds to the dimer of TtPetA with a calculated mass of 32,900 Da (for the peptide component). The second peak was 20 kDa, which corresponds to the monomer with a calculated mass of 16,450 Da. The first peak was colorless, while the second peak had a dark-brown color (Fig. 1b). Both peaks were identified as TtPetA by means of immunoblotting (Fig. 1c and Supplementary Fig. 1). The position of the second peak of the elution profile monitored with absorbance at 280 nm (*A*_280_) was slightly off from the peak monitored with *A*_380_. This may indicate that the second elution peak contained not only colored TtPetA but also colorless TtPetA.

The UV–visible spectra of TtPetA indicate that the colorless sample showed only a single absorption peak at around 280 nm (Fig. 1d), which was due to aromatic residues. This implies that this fraction had no iron-sulfur cluster. On the other hand, the monomer fraction showed absorptions in the visible region due to the iron-sulfur cluster, in addition to a peak at 280 nm. The spectrum of the brown-colored sample also indicates that the purified sample was in the reduced state, according to the comparisons with artificially reduced and oxidized samples (Supplementary Fig. 2a). The redox potential of the monomer fraction was determined to be 320 ± 4 mV (Supplementary Fig. 2b). This value is typical for a Rieske protein of the cytochrome *bc*_1_ complex^40,41^.

X-ray fluorescence spectra of SEC-purified samples revealed that the colorless TtPetA contains zinc, while only a trace amount of iron was detected (Supplementary Fig. 3). Meanwhile, the colored TtPetA contains both iron and zinc. The ratio between iron and zinc is 0.56:0.44. The ratio of iron of the brown-colored sample was increased to be 0.90:0.10 after further purification by the cation exchange chromatography. This suggests that ~80% of brown-colored sample is in the [2Fe-2S] containing form after purification, by assuming that all iron and zinc are bound in the cluster binding site and the sites for all proteins are occupied with [2Fe-2S] or a zinc ion. It should be noted, however, that the former assumption is not appropriate if the metal can be bound nonspecifically on the protein surface. The latter assumption is also inappropriate if complete apo-proteins coexist in the samples.

An in vitro reconstitution experiment demonstrated that the [2Fe-2S] cluster can actually be reconstituted into the purified sample of colorless-TtPetA even under a mild condition (Supplementary Fig. 4a). As for the stability of the [2Fe-2S] cluster, the brown color of the sample was not faded by the incubation for one week even in the presence of 10 mM zinc chloride (Supplementary Fig. 4b). It indicates that the [2Fe-2S] cluster of TtPetA were not replaced with zinc. It has recently been pointed out for several proteins that iron-sulfur clusters originally bound in the proteins may have been replaced by zinc ions during the purification processes^42,43^. However, the present result suggests that such replacement cannot occur in the case of TtPetA.

The stabilizing effect by the containing metals was examined with a protease degradation experiment. The cleavage of the Zn-containing sample with the protease was promoted in the presence of 10 mM diethylenetriaminepentaacetic acid (DTPA) (Supplementary Fig. 5a). In contrast, the cleavage of the [2Fe-2S]-containing sample showed no clear effect of DTPA addition (Supplementary Fig. 5b).

### Crystal structure of the Zn-containing form

**Fig. 2:**
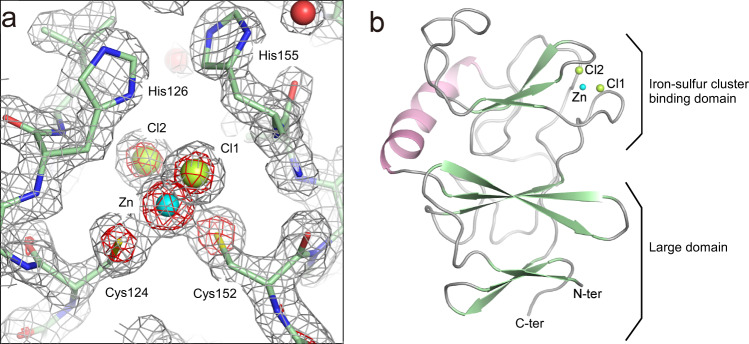
Structure of Zn-TtPetA. **a** A sigma-A-weighted 2*F*_o_–*F*_c_ map around the cluster-binding site is contoured at the 1.5σ and 5σ levels as gray and red meshes. **b** A ribbon representation of the crystal structure, where α helices, β strands and others are colored in pink, green and gray, respectively. A zinc ion in the cluster-binding site is represented as a cyan sphere, while chloride ions are shown as yellow-green spheres.

Diffraction data of the colorless TtPetA were collected at 1.7 Å resolution. The space group of crystals was *P*2_1_ with unit-cell parameters of *a* = 32.60 Å, *b* = 98.71 Å, *c* = 51.05 Å, *β* = 90.03°. Crystallographic statistics of the diffraction data are listed in Supplementary Table 1. The electron density map by Zn-SAD was of sufficient quality for auto-tracing after a density modification process. Two monomers were observed in an asymmetric unit. The final electron density map at 1.7 Å resolution was sufficient for elucidation of the structural details, such as conformations of the side chains and positions of waters and ions (Fig. 2a, Table 1 and Supplementary Data 1). An MX_2_-type compound appeared to bind in the cluster-binding site instead of the [2Fe-2S] type iron-sulfur cluster, judging from the electron density. We assigned the electron density to ZnCl_2_ based on evidence described in the next section. The structural refinement for this assignment, in which occupancies of all of these atoms were set to be 1.0, yielded reasonable *B*-factors for ZnCl_2_ and the coordination atoms, supporting the correctness of the arrangement. (Supplementary Table 2). TtPetA consists of two domains, an iron-sulfur cluster-binding domain and a large domain (Fig. 2b). The two domains are linked with loops and a helix (Fig. 2b). Such a domain structure is common for a Rieske protein of the cytochrome *bc*_1_ complex^34–37,44,45^.

**Table 1:** Refinement statistics.

Data set	Zn-TtPetA	[2Fe-2S]-TtPetA
Wavelength (Å)	1.28	1.00
Resolution range (Å)	50−1.70	50−1.79
Twin fraction	0.256	0
Constructed residues	292	292
Water molecules	145	146
Heterogens	2 × Zn^2+^, 4 × Cl^−^, 6 × SO_4_^2−^, 2 × glycerol	2 × [2Fe-2S], 1 × Cl^−^, 4 × SO_4_^2−^, 3 × glycerol
Total atoms	2465	2465
*R*_work_^a^*/R*_free_^b^ (%)	20.4/23.4	20.7/23.9
Average *B*-factor (Å^2^)		
protein	15.1	17.0
ZnCl_2_ or [2Fe-2S]	15.5	16.5
waters	19.1	22.2
other heterogens	50.5	46.0
Rmsd		
bond (Å)	0.012	0.015
angle (°)	1.6	1.8
Ramachandran plot (%)		
favored	95.83	96.53
allowed	4.17	3.47
outliers	0	0
PDB code	7YR9	7YRA

^a^*R*_work_ = Σ_hkl_||*F*_obs_| − |*F*_calc_||/Σ_hkl_|*F*_obs_|.

^b^*R*_free_ was calculated by using the 5% of the reflections that were not included in the refinement as a test set.

### Confirmation of elements in the cluster-binding site

**Fig. 3:**
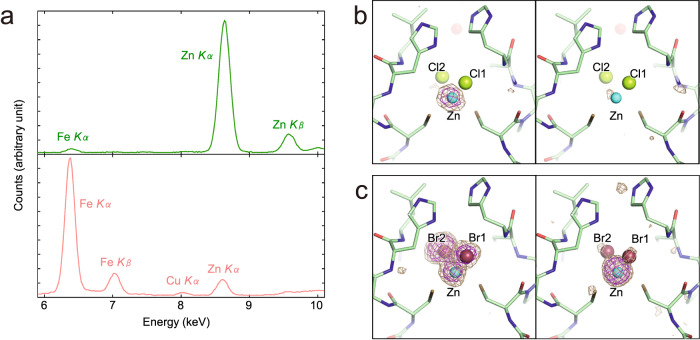
Identification of elements in the cluster-binding site. **a** The X-ray fluorescence spectra of TtPetA. The upper spectrum in green is for the immature form, while the lower one in pink is for the mature form. **b** An anomalous difference Fourier map from data collected with X-rays of *λ* = 1.28 Å is shown at the 3σ and 6σ levels as beige and magenta meshes in the left panel. A map calculated from data collected with X-rays of *λ* = 1.29 Å is shown in the right panel. **c** An anomalous difference Fourier map of a Br-soaked crystal (*λ* = 0.91 Å) is shown at the 3σ and 6σ levels as beige and magenta meshes in the left panel. A map calculated from data collected with X-rays of *λ* = 0.93 Å is shown in the right panel.

The X-ray fluorescence spectrum of the crystal of Zn-TtPetA indicates that the crystal also contains zinc (Fig. 3a) as well as the solution described above. The ratio between iron and zinc is 0.04:0.96. This corresponds to [2Fe-2S]:ZnCl_2_ = 0.02:0.98, assuming that iron and zinc are exclusively contained in the cluster binding site. On the other hand, the ratio between iron and zinc is 0.95:0.05 for the crystal of [2Fe-2S]-TtPetA, corresponding [2Fe-2S]:ZnCl_2_ = 0.90:0.10.

Because the X-ray fluorescence spectra both in solution and crystal samples indicated the presence of zinc, we attempted to determine the actual element bound in the cluster-binding site by analyzing the anomalous difference Fourier maps around the absorption edge of zinc at a wavelength of 1.28 Å (Fig. 3b). A strong electron density at the ~15σ level was observed just at the center of the cluster-binding site on the anomalous difference Fourier map of data collected at 1.28 Å, in which the *f*” value of zinc was a relatively high 3.9e^−^. On the other hand, no such densities were observed in the map of data collected at 1.29 Å, where the *f*” value of zinc was only 0.5e^−^. These observations provided confirmation that the element in the cluster-binding sites was actually zinc.

In order to confirm the element of the coordinated ions of zinc, we further collected two additional diffraction data sets from a crystal soaked in solution containing bromide ions (Supplementary Table 3). One data set was measured using X-rays of 0.91 Å, where the *f*” value of bromine was 3.7e^−^, while another data set was measured using X-rays of 0.93 Å, where the *f*” value of bromine was 0.5e^−^. Three strong densities at a level of more than 10σ were observed at the positions of the ions in the anomalous difference Fourier map of data collected at 0.91 Å (Fig. 3c). Two of these three densities disappeared in the anomalous difference Fourier map of data collected at 0.93 Å. The peak appearing on both maps is for zinc, which had an *f*” value of 2.3e^−^ even at a wavelength of 0.93 Å.

### Geometry around the ZnCl_2_ cluster

The zinc ion has a tetra-coordination manner with two chloride ions in addition to two cysteine residues in the cluster-binding site of Zn-TtPetA (Fig. 2a). The bond length of Zn−Cl and the angle of Cl−Zn−Cl are 2.21–2.51 Å and 104.2–107.1°, respectively. Although zinc ions have been observed in many protein structures, we found ZnCl_2_ only in the pH-sensitive chaperone ERp44, where ZnCl_2_ binds at the interface of two protomers^46^. The zinc ion takes penta-coordination in ERp44, in which the bond length of Zn−Cl and the angle of Cl−Zn−Cl are 2.80–3.11 Å and 60.7–63.2°, respectively. On the other hand, it has been reported that ZnCl_2_ in complex with alcohol, which takes a tetra-coordination, has similar structures with ours^47^. These facts may indicate that the geometrical parameters of ZnCl_2_ are largely influenced by the coordination number of the zinc ion. However, a previous paper reported that the distance between the zinc ion and the coordinating atom is not much affected by the coordination number, even though zinc ions take coordination numbers of 4–6^48^.

### Structural differences from the [2Fe-2S] containing form

**Fig. 4:**
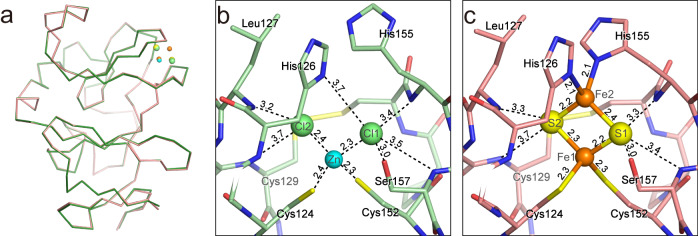
Structural differences between the Zn- and [2Fe-2S]-containing forms. **a** Superimposition of the two forms. The Cα models of the Zn- and [2Fe-2S]-containing forms are colored in green and pale red, respectively. **b** Close-up view around the cluster-binding site for the Zn-containing form. Putative hydrogen bonds and salt bridges around the ions are shown as dashed lines in black. Interatomic distances in Å units are the average value of two chains, and are indicated with dashed lines. **c** Close-up view for the [2Fe-2S]-containing form.

For a detailed comparison, the structure of the [2Fe-2S]-TtPetA was determined at 1.8 Å resolution by the molecular replacement (MR) method from the structure of Zn-TtPetA (Supplementary Fig. 6 and Supplementary Data 2). The crystallographic parameters and refinement statistics of the [2Fe-2S]-TtPetA are listed in Supplementary Table 1 and Table 1. The structure is well superimposed on those for homologous proteins of other species, especially with bacterial ones (Supplementary Fig. 7). Superimposition of [2Fe-2S]-TtPetA and Zn-TtPetA provided a root mean square deviation (rmsd) value of 0.25 Å for all atoms, indicating that there are no large differences between the two forms (Fig. 4a). This implies that conformations even in the cluster-binding sub-domain are already formed before the maturation.

The Zn, Cl1 and Cl2 atoms in the Zn-TtPetA locate at almost the same positions as Fe1, S1 and S2 in the [2Fe-2S]-PetA (Fig. 4b, c). There is no atom at the position corresponding to Fe2. Probably due to the vacancy at this position, the side chain of His155 takes a different conformation mainly by a rotation around the Cβ−Cγ bond. Accordingly, the side chain of His155 has no interactions with ZnCl_2_. The zinc ion of ZnCl_2_ interacts with Sγ of Cys124 and Cys152 with distances of 2.4 Å and 2.3 Å, respectively. Cl1 interacts with Nδ1 of His126, N of His155 and N of Ser157 with distances of 3.4–3.7 Å. In addition, Cl1 interacts with Oγ of Ser157 with a distance of 3.0 Å. On the other hand, Cl2 interacts with the main chain N atom of Leu127 and N atom of Cys129 with distances of 3.2 Å and 3.7 Å, respectively.

### Oligomer structure of Zn-TtPetA

The molecular mass of Zn-TtPetA was measured with SEC-multi-angle light scattering (MALS) analysis. The elution profile shows one major peak and one minor peak (Supplementary Fig. 8a), even though the first colorless fraction of the SEC purification was used in the analyses. The molecular masses for the major and minor peaks are estimated to be 32.9 kDa and 18.9 kDa, respectively, indicating that the first SEC peak actually corresponds to dimeric TtPetA. In addition, the second peak reappeared despite the application of a sample of the first peak of the SEC purification (Supplementary Fig. 8a), implying that the monomer and dimer of Zn-TtPetA are in equilibrium in solution.

**Fig. 5:**
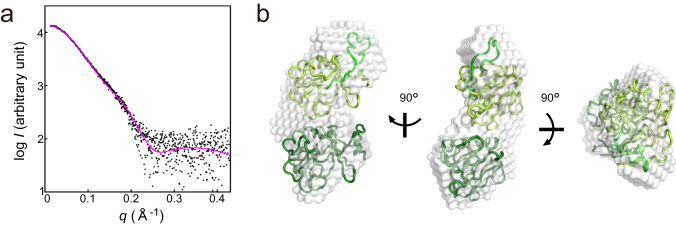
SAXS analysis of Zn-TtPetA. **a** X-ray scattering profile. Experimental values are plotted as black dots. The calculated profiles for a model derived from a crystal packing with some structural changes in the C-terminus (Model II-C) is as the magenta line. **b** Envelope model from the SAXS data. The dimer model is fitted where for two protomers are shown in green and yellow green. The C-terminal region of the upper protomer, which is modified from the crystal structure, is highlighted in light green.

In order to obtain more detailed information for the dimeric structure of Zn-TtPetA in solution, a SEC-SAXS experiment was performed. The radius of gyration (*R*_g_) remained constant during the elution of the first peak, indicating that the sample contained in the first elution peak is highly uniform in shape as well as the size of the sample (Supplementary Fig. 8b). As a result, an X-ray scattering profile with sufficient quality for the SAXS analysis was obtained (Fig. 5a). An ab initio model derived from the SAXS data is the elongated shape with an approximate dimension of 80 × 40 × 40 Å^3^ with slight bend at the middle of the model (Fig. 5b).

To construct the atomic dimer structure in solution, three putative models (Models I, II, and III) were generated from the crystal packing by the PISA server^49^ (Supplementary Fig. 9a–c). The calculated scattering curve of Model II fit to the experimental scattering curve and the pair distribution function *P*(*r*) better than those of the other models (Supplementary Fig. 6c and d). Actually, the *χ*^2^ value for Model II (*χ*^2^ = 0.062) is notably smaller than those for Model I (*χ*^2^ = 0.213) and Model III (*χ*^2^ = 0.203). Therefore, Model II is the most plausible one among the three models. The Model II has no twofold axis between the two protomers, while the two is related by a translational symmetry. Therefore, two protomers are non-equivalent in the dimer model (Supplementary Fig. 9b). Model II can be well superimposed onto the ab initio model, while some small discrepancies were found (Fig. 5b). The modification of the conformation at the C-terminal region of a protomer improved the superimposition even without other changes (Fig. 5b). The *χ*^2^ value for the modified model (Model II-C) was decreased to be 0.056. The altered conformation of the C-terminus for one protomer may explain the propensity of the protomer to form dimers but not much larger oligomers. In Model II-C as well as Model II, the inter-protomer interaction has a contact area of ~300 Å^2^ according to the PISA server (Supplementary Fig. 9d). The Δ*G* of dimer formation was estimated to be −22 kJ/mol, or ~9 times the value of *kT* at room temperature. The relatively weak interaction between the protomers can be related to the fact that the dimer is in equilibrium with the monomer in solution.

On the interfaces of Model II, many conserved residues are involved in the interactions between the two protomers according to the analysis with the PISA server. A hydrophobic patch consisting of residues around Cys129 and Cys154 forming a disulfide bond such as Leu127, Gly128 Ser130, Pro153 and His155 interacts with another hydrophobic patch consisting of residues, Pro79, Glu80, Leu82, Ala83, Pro85, Asn108, Pro109, Thr110, and Gln116 of another protomer (Supplementary Fig. 9d and e). The interaction seems to be enhanced with hydrogen bonds mediated by water molecules observed in the crystal structure. The residues of two patches are highly conserved in bacterial homologs (Supplementary Fig. 7a). In addition to the hydrophobic interface described above, a salt-bridge between Lys66 of a protomer and Asp185 of another protomer is formed (Supplementary Fig. 9d). These residues are also highly conserved among the homologous proteins. Therefore, it is possible to form dimeric structures, at least in bacterial homologous proteins.

## Discussion

The structure of the Zn-containing form of a Rieske protein, in which ZnCl_2_ binds in the cluster-binding site, was determined. Structural differences from the [2Fe-2S]-containing form were observed exclusively in the vicinity of the binding site, whereas large differences had been reported in the case of a FeMoco protein having a larger cofactor^50^. Since the Zn-containing form is as stable as the [2Fe-2S] form, it is implied that ZnCl_2_ may act as a temporary stabilizer until [2Fe-2S] insertion. Which metal ions are actually bound in vivo should be discussed, while the zinc is the most plausible candidate because of the amount in bacterial cells. On the other hand, it has been reported that a state in which only one iron binds to the cluster assembly site is critical for the assembly of the [2Fe-2S] cluster in the scaffold protein IscU^30–33^. Therefore, iron ions can also be promising candidates as inherently binding metal ion. However, unlike the case of IscU, which requires iron ions as a component of the [2Fe-2S] cluster, there seems to be no functional necessity for PetA to bind iron ions. Furthermore, it has been pointed out that zinc ion may have roles in the inhibition of oxidation of cysteine residues and the stabilization of the conformation of eukaryotic ISCU and bacterial IscU^30–33^. Even in PetA, the removal of zinc from Zn-TtPetA was demonstrated to decrease resistance to protease digestion as shown before (Supplementary Fig. 5). Because zinc and iron ions have similar distances and coordination numbers of metal ligands^4^, the Zn-containing structure determined in this study might represent an immature form appearing before the maturation of iron-sulfur cluster-containing proteins.

The coordination of some metal ion to the immature form of iron-sulfur cluster-containing proteins may be important to avoid inappropriate situations such as oxidation of cysteine and degradation by proteases while waiting for insertion by the Isc system. Of course, since zinc ion is one of the most abundant metal ions in cells, their binding to the cluster binding site may not be artificial and may reflect an intrinsic intracellular process. Actually, zinc ions have widely been observed in some structures of maturation factor proteins of the iron-sulfur cluster^21,23,26–28,51,52^. In addition, zinc ions were indicated to have some modulator roles in the maturation of the iron-sulfur cluster^28,53–55^. Zinc ions were also indicated to play a role in the integrity of the Rieske subunit of the cytochrome *bc*_1_ complex^56^. However, the intrinsic role of zinc ion in the maturation process has not yet been clarified. The structure of the immature Rieske protein allow us to speculate that an exchange reaction of the iron-sulfur cluster and a zinc ion may take a place between the immature protein and a component of the ISC system at the incorporation of the iron-sulfur cluster. A candidate of the [2Fe-2S] donor is [2Fe-2S]-loaded IscU. However, GrxD of the Isc system can also be a candidate as the exchange partner^57^. In fact, *T. tepidum* has also a homologous protein of GrxD (Uniprot ID: A0A6I6DY12) with an identity to GrxD from *E. coli* of 53%. However, further studies are needed to determine what the direct [2Fe-2S] donor to PetA actually is. Nevertheless, the presence of such metal-cluster exchange process in the insertion step would be acceptable to the maturation mechanisms that have been proposed so far^30,51,58^. It has been reported that a zinc ion bound to IscU can be released by IscS^32^, although the zinc ion must be replaced by an iron ion to allow the assembly of the iron-sulfur cluster^30^.

The oligomeric states differ from each other despite the small structural differences at the binding site. This may be related to differences in the structure of the cluster binding sites, according to the constructed dimer model by the SAXS analysis. However, it is difficult to provide a clear explanation as to why only the Zn-containing form dimerizes based on the models. One possible explanation is His155, a ligand residue of the [2Fe-2S] cluster, is involved in the change of the oligomeric state. Indeed, the conformation of His155 is different between the dimeric and monomeric forms (Fig. 4b, c). This residue is actually located at the interface between the two protomers of our proposed dimer structure. On the other hand, [2Fe-2S]-TtPetA forms a similar dimer in the crystal with very high protein concentration, even though the conformation of His155 in [2Fe-2S]-TtPetA is not suitable for dimer formation. This may imply that other interactions on the interface also contribute to some extent to dimer formation. What then is the role of dimer formation in the immature form? It is possible that the dimer stabilizes immature proteins to avoid degradation by proteases, like metal coordination. Indeed, the SAXS model indicates that the structure of the C-terminal region can be fluctuate in solution. Even in this case, the [2Fe-2S] insertion would not be inhibited by the dimer formation, because the monomer and dimer are in equilibrium. However, it is difficult to provide any definitive conclusion about the physiological role of dimer formation of the Zn-containing form from this structural study.

The presence of zinc ion in the cluster binding site of TtPetA suggests that zinc ions may play various roles in the insertion step of the iron-sulfur cluster. However, further studies will be needed to clarify the insertion mechanism, including elucidation of the intermediate structures upon the exchange. The knowledge gained in this study, as well as the purified sample of the putative immature form of the iron-sulfur protein, will be useful for future studies.

## Materials and methods

### Construction, expression and purification

The gene for the Rieske subunit of the cytochrome *bc*_1_ complex of *T. tepidum* optimized for *E. coli* expression was prepared by artificial gene synthesis (GeneArt®; Thermo Fisher Scientific, Waltham, MA). A region of the gene corresponding to the soluble domain (residues Pro49−Ala197) was ligated into pET21a (Novagen, Madison, WI). The recombinant TtPetA has a His-tag at the C-terminus.

For overexpression, transformed *E. coli* Rosetta (DE3) (Novagen) was grown at 37 °C in LB medium containing 50 μg/mL FeSO_4_·7H_2_O, 50 μg/mL ampicillin and 50 μg/mL chloramphenicol. The protein production was induced with 1.0 mM isopropyl-β-D-thiogalactopyranoside at 22 °C after reaching an OD_600_ = 0.6. Cultured cells were collected after 24 h of induction by centrifugation (9200 × *g*) for 10 min at 4 °C.

The cells were suspended in buffer A [10 mM Tris-HCl (pH 7.6), 150 mM NaCl] and pelleted again by centrifugation. The pellet was suspended in buffer A containing EDTA-free protease-inhibitor cocktail (Nacalai Tesque, Kyoto, Japan) and 1 mg/mL lysozyme. The cells in the suspension were broken by sonication after incubation for 30 min on ice. The cell lysate was centrifuged (14,500 × *g*) for 30 min at 4 °C, and the supernatant was loaded onto an Ni-NTA affinity column (Quiagen, Germantown, MD) equilibrated with buffer A. Proteins were eluted with buffer A supplemented with 500 mM imidazole. Immediately after elution, the solution was replaced by buffer B [10 mM Tris-HCl (pH 7.6)] via ultra-filtration with Amicon Ultra 10k (Merck Millipore, Burlington, MA). The His-tag sequence was truncated with porcine pancreas carboxypeptidase B (Sigma-Aldrich, St. Louis, MO) or subtilisin (Sigma-Aldrich) at a 1:100 ratio to TtPetA. After removal of the His-tag, the protein was purified by SEC using a HiLoad 16/60 Superdex 75 column (GE Healthcare, Chicago, IL) equilibrated and eluted with buffer A. The void volume of the SEC column was determined with blue dextran. The molecular weights were estimated with the standard marker mixture for low molecular weight proteins (GE Healthcare).

For the colored fraction, further purification was carried out. The buffer was exchanged to buffer B and the protein was incubated with 10 mM EDTA overnight at 20 °C. The sample was applied to anion-exchange chromatography using a HiLoad 26/16 Sp Sepharose column (GE Healthcare) equilibrated with 20 mM MES (pH 6.5) and eluted with a gradient from 0 to 500 mM NaCl. The main peak fractions were collected and the buffer was replaced by buffer B. Both purified proteins were concentrated to 14 mg/mL and frozen in liquid nitrogen.

Anti-TtPetA antibody was prepared from rabbit immunized with a peptide corresponding to an N-terminal region of the protein by a custom service (Eurofins Genetics, Tokyo). Proteins were separated with the Tris-Tricine SDS-PAGE (15%) and the bands for TtPetA were visualized by a WesternBreeze chromogenic kit (Thermo Fisher Scientific, Waltham, MA) after transferring onto an Immobilon-P PVDF membrane with a pore size of 0.2 μm (Merck Millipore).

UV–visible absorption spectra of the purified samples were measured using a V-630 spectrometer (JASCO, Tokyo, Japan) in a range from 250 to 650 nm at a room temperature of ~20 °C. The mature TtPetA was dissolved in 10 mM Tris-HCl buffer (pH 7.6). Oxidation or reduction of the protein was completed by adding 5 mM K_4_Fe(CN)_6_ or 5 mM DTT for 1 h. The reagents were removed by repeated concentration and dilution with ultrafiltration.

### Determination of the redox potential

The redox potential of [2Fe-2S]-TtPetA was measured by a spectroelectrochemical titration method using the Fe(CN)_6_^4-^/Fe(CN)_6_^3-^ (ferrocyanide/ferricyanide) redox couple^59^. The protein concentration was 0.3 mg/mL in 50 mM K-phosphate buffer (pH 7.0) and 100 mM K_4_[Fe(CN)_6_]. Absorption spectra were measured using V-630 spectrometer (JASCO) with an increment of K_3_[Fe(CN)_6_]. The absorbance at 500 nm was used in the determination of the redox potential. The solution potentials (*E*_s_) were calculated from the Nernst equation,

$${E}_{{{{{{\rm{s}}}}}}}={E}^{{{{{{\rm{o}}}}}}{\prime} }+\frac{{RT}}{F}{{{{\mathrm{ln}}}}}\frac{\left[{{{{{{\rm{K}}}}}}}_{3}[{{{{{{\rm{Fe}}}}}}({{{{{\rm{CN}}}}}})}_{6}]\right]}{\left[{{{{{{\rm{K}}}}}}}_{4}[{{{{{\rm{Fe}}}}}}{({{{{{\rm{CN}}}}}})}_{6}]\right]}$$

where *E*^o’^ is the standard redox potential of the ferrocyanide/ferricyanide system in 50 mM K-phosphate buffer (pH 7.0)^59^, and *R*, *T* and *F* are the molar gas constant, absolute temperature and Faraday constant, respectively. The redox potential of TtPetA was derived from the ln{(*A*_red_ – *A*_obs_)/(*A*_obs_ – *A*_ox_)} vs *E*_s_ plot (Supplementary Fig. 2b). The error of the redox potential was estimated as the error of the *y*-intercept in the linear least squares method.

### X-ray fluorescence spectroscopic analysis

X-ray fluorescence spectra were measured at BL41XU of SPring-8 (Hyogo, Japan) with a FAST SDD silicon drift detector (Amptec, Bedford, MA). The wavelength of the incident X-rays was 1.00 Å. Solution samples were air-dried prior to the measurement, while crystalline samples were frozen in liquid nitrogen. All samples were cooled at −173 °C with a nitrogen-gas stream during the measurement. In order to estimate the concentration ratio of iron and zinc in the samples, we also measured reference spectra of solutions containing various concentrations (5.0/5.0/10.0, 10.0/10.0/10.0 and 20.0/20.0/10.0 mM) of ZnSO_4_/FeSO_4_/NiSO_4_ and 50% glycerol.

### Reconstitution and replacement of binding metals

The binding ability of the [2Fe-2S] cluster was examined with an in vitro method. A solution containing 0.3 mg/mL Zn-TtPetA, 70 μM iron(III) chloride, 70 μM sodium sulfide, 1.0 mM dithiothreitol, 50 mM Tris-HCl (pH 8.0) and 150 mM sodium chloride was incubated for one day at 20 °C. Buffer exchange and concentration were done by ultra-filtration with Amicon Ultra 10k (Merck Millipore). The UV–visible absorption spectra were measured by the cuvette mode of a NanoPhotometer-NP80 spectrometer (Implen, Munich, Germany). The replacement of the [2Fe-2S] cluster in the purified [2Fe-2S]-TtPetA by zinc ions was also investigated in vitro. A solution containing 1.0 mg/mL [2Fe-2S]-TtPetA, 50 mM Tris-HCl (pH 7.6) and 150 mM sodium chloride was incubated in the presence of 0, 1.0, or 10 mM zinc chloride at 20 °C. The solutions were sampled at 0, 1, 3, and 7 days after starting the incubation, and the UV–visible absorption spectra were measured. Absorptions at 400 nm, which is an isosbestic point of reduced and oxidized forms were plotted as a function of incubation time, because the sample were oxidized during the incubation.

### Protease degradation assay

0.5 mg/mL Zn-TtPetA or [2Fe-2S]-TtPetA was incubated with 5 μg/mL subtilisin in a solution (50 mM Tris-HCl pH 7.6, 150 mM sodium chloride, and 200 mM ammonium sulfate) in the presence or absence of 10 mM DTPA at 35 °C. The solutions were sampled at 0, 0.25, 1, 2, and 5 days after starting digestion. The samples were analyzed with SDS-PAGE using 16.5% Tris-Tricine gels.

### Crystallization and crystallographic data collections

Crystals for both the immature and mature forms of TtPetA were obtained by the sitting drop vapor diffusion method at 20 °C in a solution that was a 1:1 mixture of protein solution [~10 mg/mL protein in 50 mM MES (pH 6.5)] and reservoir solution [100 mM Na-acetate (pH 4.6), 1.6 M (NH_4_)_2_SO_4_ and 20% glycerol]. Crystals were flash-cooled with a nitrogen-gas stream at −173 °C and stored in liquid nitrogen until used in experiments.

Diffraction data were also collected at BL41XU of SPring-8. The X-ray wavelength was set to 1.28 Å and 1.29 Å for the immature TtPetA crystals (Supplementary Table 1). In addition, diffraction data of the mature TtPetA crystals were collected using X-rays of 1.00 Å, 1.74 Å, and 1.75 Å in wavelength (Supplementary Table 1). Two data sets were also measured using X-rays of 0.91 Å and 0.93 Å from an immature TtPetA crystal soaked in a solution containing 200 mM NaBr in addition to the original crystallization condition for 3 days (Supplementary Table 3). The data were processed with the XDS (vJan.31.2020) program^60^.

### Crystal structure determination

The initial experimental phase of the immature TtPetA data was obtained with the single-wavelength anomalous dispersion (SAD) method by the anomalous scattering of zinc. The Zn-SAD phasing was carried out using the data collected at 1.28 Å with the SOLVE (v2.13) program^61^, and subsequent density modification was performed with the RESOLVE (v2.15) program^62^ in the PHENIX (v1.14-3260) suite^63^. Two zinc sites were found with the SOLVE program giving a value of figure of merit (FOM) of 0.24. The FOM value was improved to be 0.60 with the density modification process. The model was constructed by autotracing with the ARP/wARP (v8.0) program^64^ in the CCP4 (v7.0) suite^65^ and manually improved with the COOT (v0.8.9.2) program^66^. While the data showed twinning (twin law: −*h*, −*k*, *l*) according to the CTRUNCATE program in the CCP4 suite^65^, the model building was successfully completed without consideration of twinning. The refinement calculations were performed with the CNS (v1.3) program^67^. In the course of the refinement calculation process, twinning was taken into account. The twin fraction was refined to 0.256. The final *R*_work_ and *R*_free_ factors at 1.7 Å resolution were 20.4% and 23.4%, respectively. The refinement statistics are listed in Table 1. As for the mature TtPetA data, the crystal structure was solved by the MR method with the MOLREP (11.7.02) program^68^ in the CCP4 suite using the coordinates of the refined immature TtPetA structure as a search model. The wRfac and Score values for the solution was 0.374 and 0.750, respectively. The output structure was manually improved and refined using the COOT and CNS programs. The structures were validated with the Molprobity (v4.1) program^69^ in the PHENIX suite. No residues were in the outlier region in the Ramachandran plot for either structure. All figures for molecular models were generated using the program Pymol (v2.4.0a0)^70^.

### SEC-MALS analysis

The SEC-MALS data were measured using an Agilent 1260 infinity HPLC system (Agilent Technologies, Santa Clara, CA) equipped with Superdex 200 Increase 10/300 GL column (Cytiva, Marlborough, MA), Optilab rEX differential refractometer (Wyatt Technology, Santa Barbara, CA) and DAWN HELEOS II 8+ light scattering detector (Wyatt Technology). Approximately 100 µL of protein solution (7.3 mg/mL) was injected and was run with buffer A at a flow rate of 0.5 mL/min at 24 °C. The data was analyzed with the ASTRA 7 (v7.1.4.8) program (Wyatt Technology).

### SEC-SAXS analysis

The SEC-SAXS experiments were performed at BL38B1 of SPring-8 at 20 °C. The wavelength of the incident X-ray was 1.00 Å and the beam size was ~0.6 × 0.4 mm^2^ at sample position with a flux of 5 × 10^10^ photons/s. X-ray scattering images were measured with a PILATUS3 S 2 M detector (DECTRIS, Baden-Daettwil, Switzerland). The sample-to-detector distance was 2572 mm. The measurement system was calibrated using diffraction from silver behenate (NS470102, Nagara Science, Gifu, Japan). Approximately 200 μL of protein solution (7.0 mg/mL) was injected into a Superdex 200 increase 10/300 column (Cytiva) operated by an HPLC system (Shimadzu, Kyoto, Japan) with an elution buffer containing 10 mM Tris-HCl (pH 7.6), 150 mM sodium chloride and 5% glycerol at a flow rate of 0.3 mL/min. The sample solution was eluted into the sample cell with 0.02 mm-thick quartz glass windows and 1 mm-path length. The X-ray scattering images were collected with a data acquisition time of 3 s/frame and 1600 frames during the elution for the column volume of 24 mL. Each frame’s incident and transmitted intensity were measured during X-ray exposures with an MIC-205 ionized chambers (AVS US Inc., Lansing, NY) which were placed upstream and downstream of the sample container. The 2D images were processed to obtain 1D scattering profiles with the SAngler (v2.1.65) program^71^. The absolute scattering intensity was calibrated with water^72^. UV–visible absorption spectra of the sample in the cell were simultaneously recorded using a QEpro UV–visible spectrometer (Ocean Insight, Orlando, FL). The SEC-SAXS data were analyzed with the MOLASS (v1.0.11) program^73^. The range of the magnitude of the scattering vector *q* used for the SAXS analysis was 0.013 < *q* (=4πsin*θ*/*λ*) < 0.43 Å^−1^, where *θ* is a half of the scattering angle and *λ* is the wavelength of X-rays. The scattering curves and the pair distribution functions *P*(*r*) for dimer models were calculated with the CRYSOL and GNOM (v5.0) programs^74,75^ in the ATSAS (v3.2.1) suite^76^. The models were evaluated with the *χ*^2^ value. The ab initio model was obtained from the experimental scattering curve the DAMMIF program^77^ in ATSAS using the maximum dimension (*D*_max_) value of 78 Å where the experimental *P*(*r*) curve was smoothly approached to zero.

### Reporting summary

Further information on research design is available in the Nature Portfolio Reporting Summary linked to this article.

### Supplementary information

Peer Review File

Supplementary Information

Description of Additional Supplementary Files

Supplementary Data 1

Supplementary Data 2

Reporting summary

## Data Availability

The coordinates and structural factors for X-ray crystal structures of Zn-TtPetA and [2Fe-2S]-TtPetA have been deposited in the Protein Data Bank under accession numbers 7YR9 (Supplementary Data 1) and 7YRA (Supplementary Data 2), respectively. The X-ray scattering data of the small angle X-ray scattering analysis for Zn-TtPetA have been deposited in the small-angle scattering biological data bank (SASBDB) with an accession code of SASDSV5.
