# Supplementary material for: Structure of a putative immature form of a Rieske-type iron-sulfur protein in complex with zinc chloride
Source: Commun Chem. 2023 Sep 9;6:190. doi: 10.1038/s42004-023-01000-6 (PMC10492824; doi:10.1038/s42004-023-01000-6)
Supplement: Supplementary file 2 — Supplementary Information [file 42004_2023_1000_MOESM2_ESM.pdf]

Supplementary Information

Structure of a putative immature form of a Rieske-type iron-sulfur protein in complex with zinc chloride

Erika Tsutsumi[1,4], Satomi Niwa[1,4], Ryota Takeda[1], Natsuki Sakamoto[1], Kei Okatsu[1], Shuya Fukai[1], Hideo Ago[2], Satoshi Nagao[3], Hiroshi Sekiguchi[3], Kazuki Takeda[1*]

[1]Department of Chemistry, Graduate School of Science, Kyoto University, Sakyo-ku, Kyoto 606-8502, Japan

[2]RIKEN SPring-8 Center, 1-1-1 Kouto, Sayo, Hyogo, 679-5148 Japan

[3]Japan Synchrotron Radiation Research Institute (JASRI), 1-1-1 Kouto, Sayo-cho, Sayo-gun, Hyogo 679-5198, Japan

[4]These authors contributed equally

[*]Corresponding e-mail: ktakeda@kuchem.kyoto-u.ac.jp

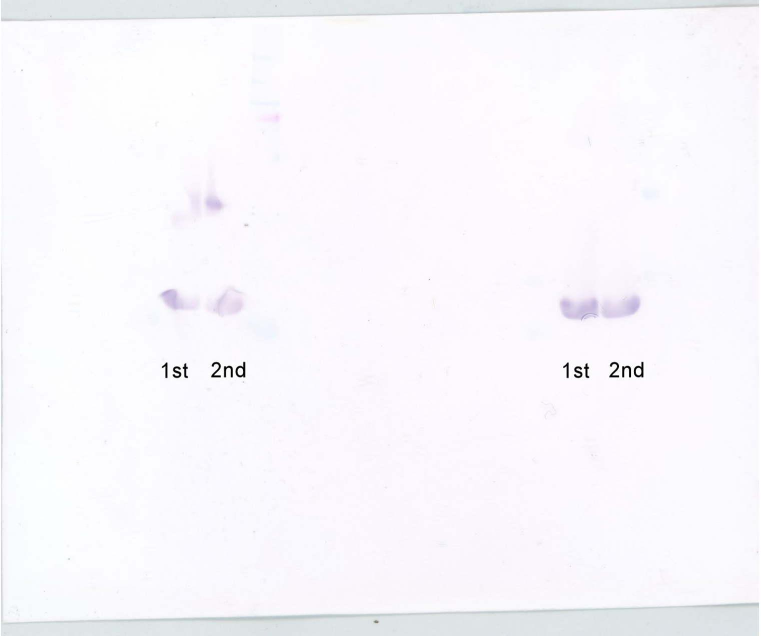

Supplementary Figure 1 : Full image of Fig. 1c.

Immunoblotting for the first and second peaks of SEC. The protein concentration in the two left lanes was one-half that of the two right lanes. In Fig. 1c, the two right lanes are shown.

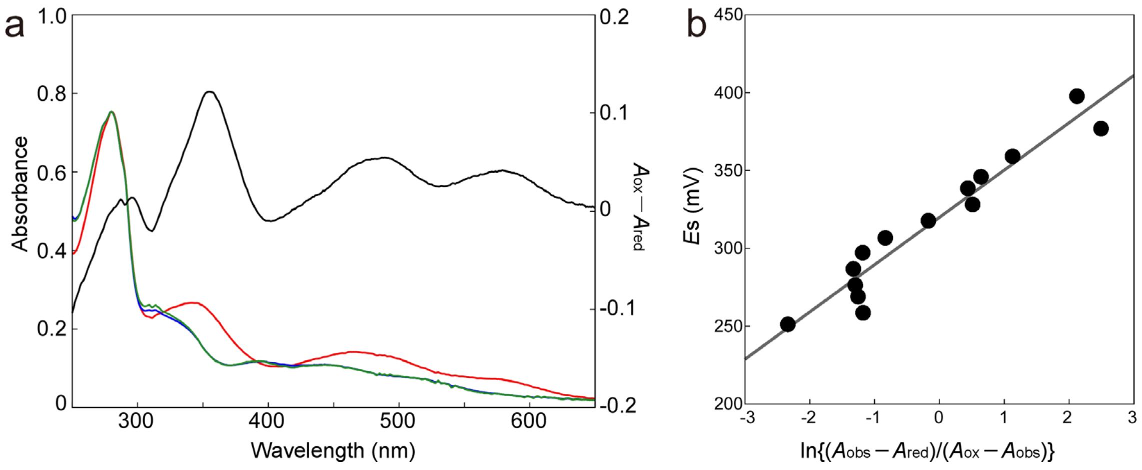

Supplementary Figure 2 : Redox properties of the purified samples.

(a) Redox changes on the absorption spectra of [2Fe-2S]-TtPetA. The UV-vis spectrum for the non-treated sample (0.7 mg/mL) is shown as a green line. The spectra for the artificially reduced and oxidized samples are shown as blue and red lines, respectively. The spectra were scaled by fitting to the absorbance at 280 nm of the non-treated sample. The difference spectrum ($A_{ox} - A_{red}$) of both states is also shown as a black line. **(b)** Redox potential analysis of [2Fe-2S]-TtPetA. The $\ln\{(A_{red} - A_{obs})/(A_{obs} - A_{ox})\}$ *vs* E_s plot from the spectroelectrochemical titration of [2Fe-2S]-TtPetA. The *y*-intercept of the line derived by the least-squares method corresponds to the redox potential.

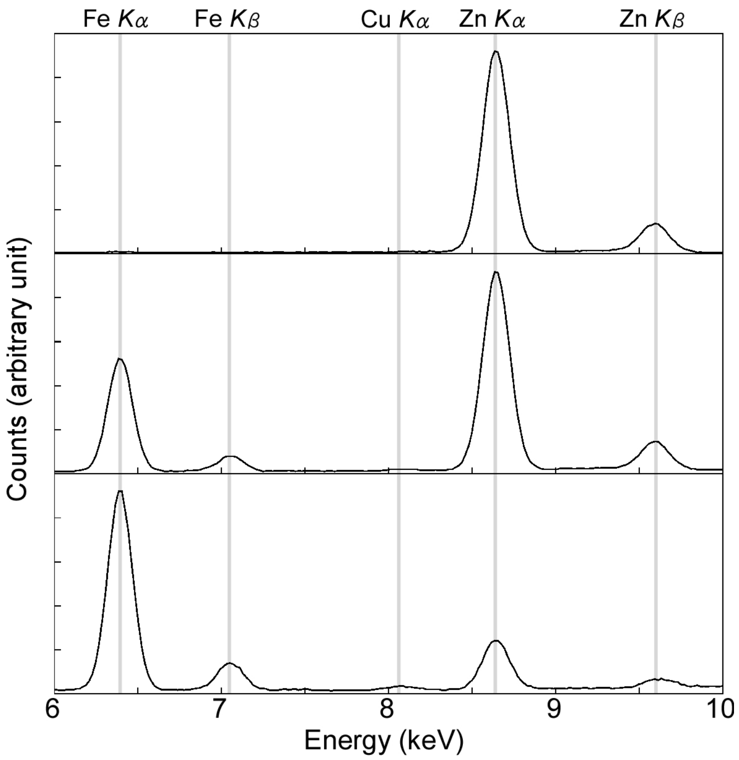

Supplementary Figure 3 : Confirmation of contained metals in solution samples. The X-ray fluorescence spectra for the first and second elution peaks of SEC are shown in the top and middle panels. That for a further purified sample of the second elution peak of SEC with the cation exchange chromatography are shown in the lower panel.

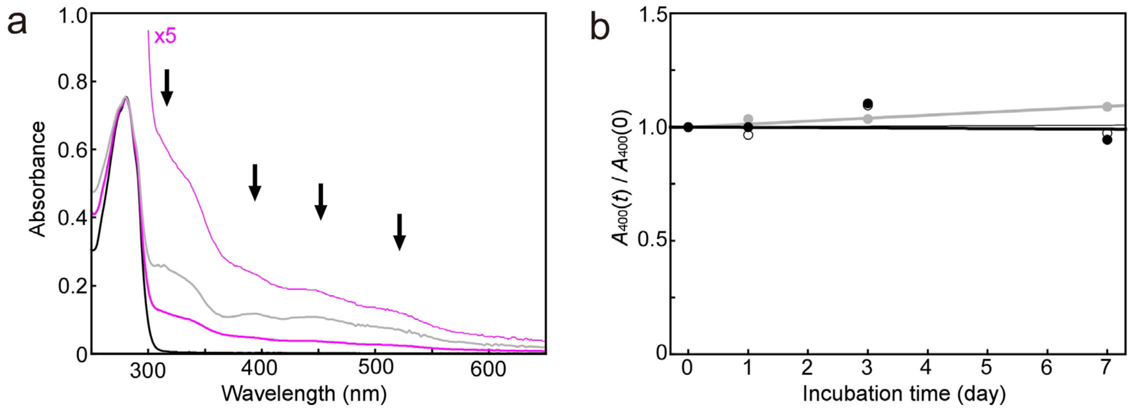

Supplementary Figure 4 : Reconstitution and replacement of binding metals

(a) *In vitro* reconstitution of the [2Fe-2S] cluster into Zn-TtPetA. The spectra for the sample incubated in the presence of $FeCl_3$/Na_2S/DTT, the sample incubated without $FeCl_3$/Na_2S/DTT (negative control) and purified [2Fe-2S]-TtPetA (identical to the spectrum of the non-treated sample in panel a) were shown as magenta, black and gray lines. The spectra were scaled by fitting to the absorbance at 280 nm of purified [2Fe-2S]-TtPetA. The characteristic peaks at ~320, 400, 450 and 520 nm are indicated by arrows. **(b)** Stability of the [2Fe-2S] cluster in the presence of $ZnCl_2$. Changes of absorbance at 400 nm (A_{400} (t) / A_{400} (t=0)) were plotted against incubation time. The changes in the presence of 0, 1.0 and 10 mM $ZnCl_2$ are plotted as open circles, gray solid circles and black solid circles, respectively. The thin black, gray and black lines were determined for each plot by the least-squares fitting of $y= ax+1$.

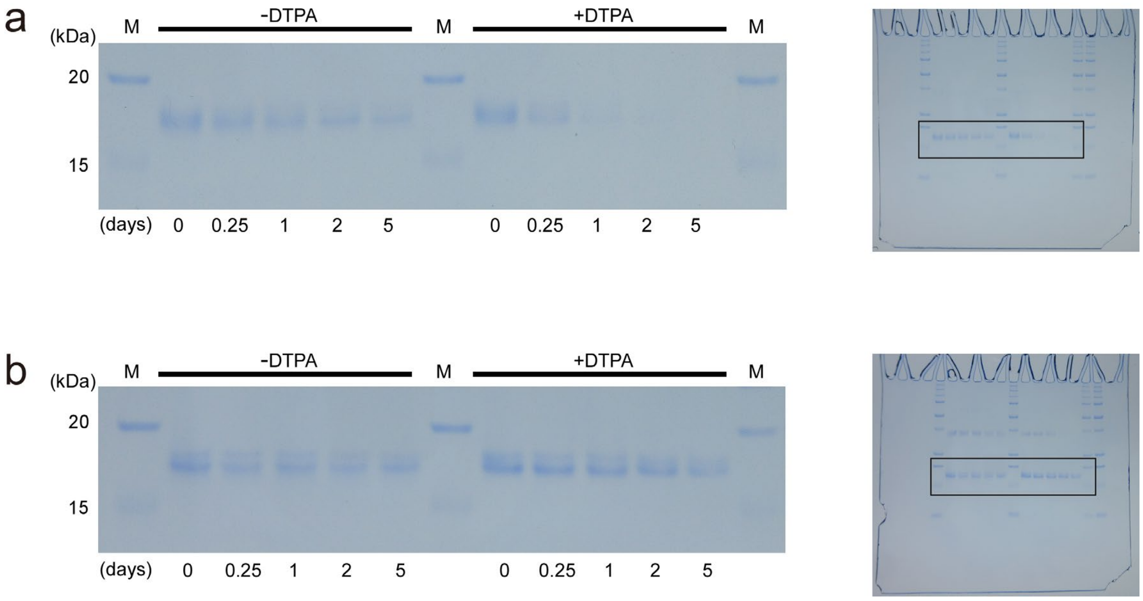

Supplementary Figure 5 : Protease degradation assay.

(a) The SDS-PAGE gel for the Zn-containing sample. The sample was incubated with subtilisin at 35 °C in the absence or presence of 10 mM DTPA, and sampled at 0, 0.25, 1, 2 and 5 days after starting digestion. A full image of the gel is shown in the right side. The trimming boundary is indicated by a box. **(b)** The result for the [2Fe-2S]-containing sample.

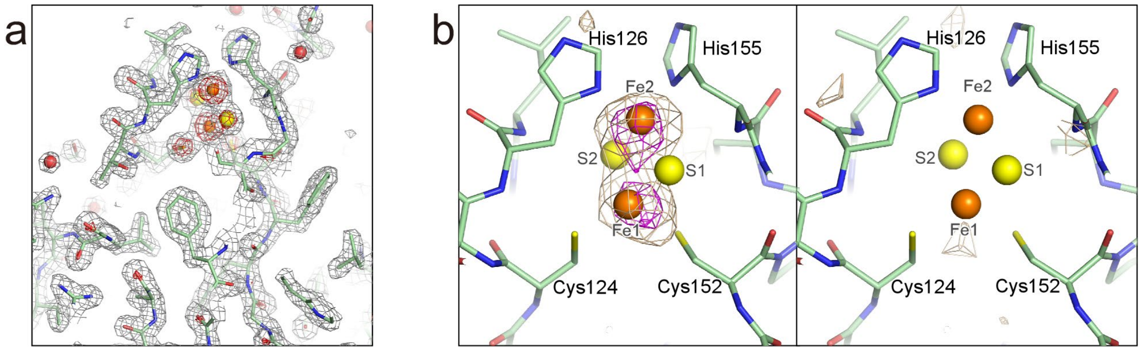

Supplementary Figure 6 : Electron density maps for [2Fe-2S]-TtPetA.

(**a**) A sigma-A-weighted $2F_o - F_c$ map is contoured at the 1.5σ and 5σ levels as gray and red meshes. (**b**) An anomalous difference Fourier map from data collected with X-rays of λ= 1.74 Å is shown at the 3σ and 6σ levels as beige and magenta meshes in the left panel. A map calculated from data collected with X-rays of λ= 1.75 Å is shown in the right panel.

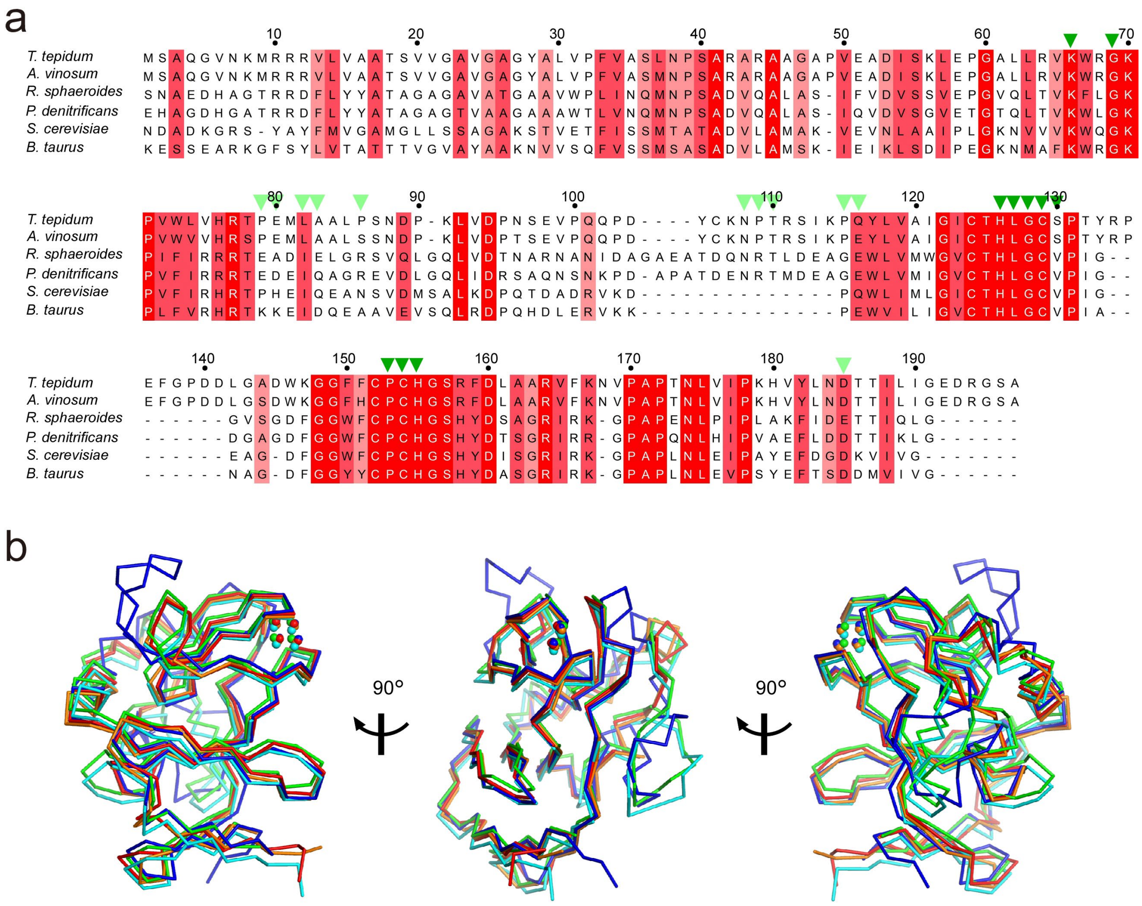

Supplementary Figure 7 : Structural comparison with homologous proteins.

(**a**) Multiple sequence alignment for the homologous proteins of TtPetA. Sequences for the proteins from *T. tepidum* (UniProt ID: D1MZ11), *A. vinosum* (O31214), *R. sphaeroides* (Q02762), *P. denitrificans* (P05417), *S. cerevisiae* (P08067), *B. taurus* (P13272) were aligned with the ClustalW program[1]. For proteins other than ThPetA, only sequences corresponding to the same region with TtPetA are presented in the alignment. Residues are shaded with red, light red, or pale red according to the degree of conservation. The residue number for TtPetA is given in the upper side of its sequence, where the start methionine is numbered as 1. Interfacing residues in one protomer are indicated by green triangles, while those for another protomer are by light green triangles. (**b**) Superimposition of the structure of TtPetA with the homologous protein. The superimpositions were performed with the DALI server[2]. Crystal structures for the Rieske domains from *T. tepidum* (IDB-ID: 7YRA), *R. sphaeroides* (2NUK)[3], *P. denitrificans*

(2YIU)[4], *S. cerevisiae* (3CX5) [5], *B. taurus* (1RIE)[6] are shown in blue, cyan, green, orange and red, respectively. The rmsd values against above structures provided by the DALI superimposition were 1.7 Å for 141 residues (Z score = 18.3), 1.8 Å for 137 residues (17.8), 1.6 Å for 125 residues (17.6) and 1.6 Å for 127 residues (17.4), respectively.

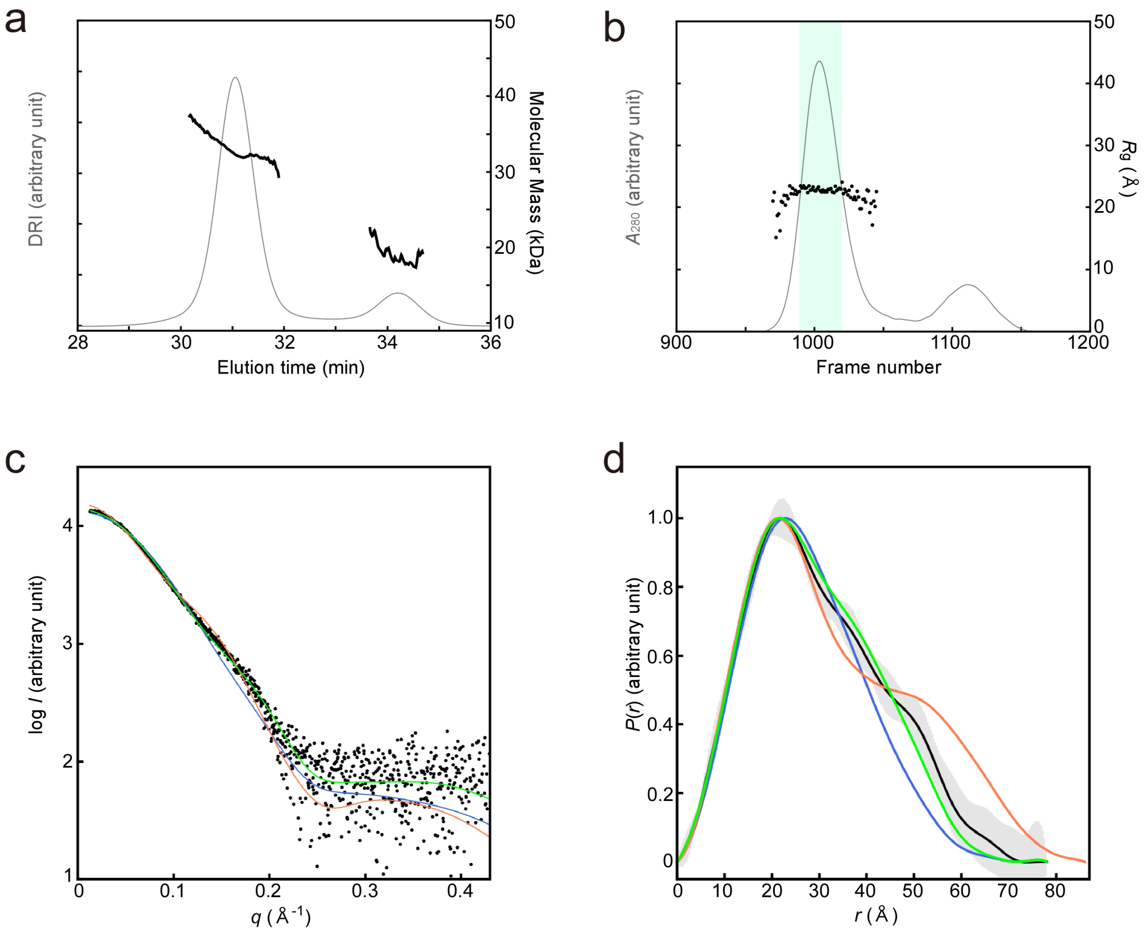

Supplementary Figure 8 : Oligomeric state analyses for the colorless sample.

(a) SEC-MALS profile. The gray line indicates the differential refractive index monitoring the existence of proteins, and the black lines indicate molecular masses. **(b)** SEC-SAXS profile. The gray line indicates UV absorbance at 280 nm for each scattering frame, which indicates the existence of proteins, and the black dots indicate R_g values for each frame. The green shade indicates the range of frames used for the SAXS analysis. **(c)** X-ray scattering profile. Experimental values are plotted as black dots. The calculated profiles for three dimer models found in the crystal are overlayed. Blue, green and orange correspond to models I (Supplementary Fig. 9a), II (Supplementary Fig. 9b) and III (Supplementary Fig. 9c), respectively. **(d)** Pair distance distribution function. The function derived from the experimental data is plotted as a black line with errors in gray. The calculated functions from the three models above are overlayed on the experimental function.

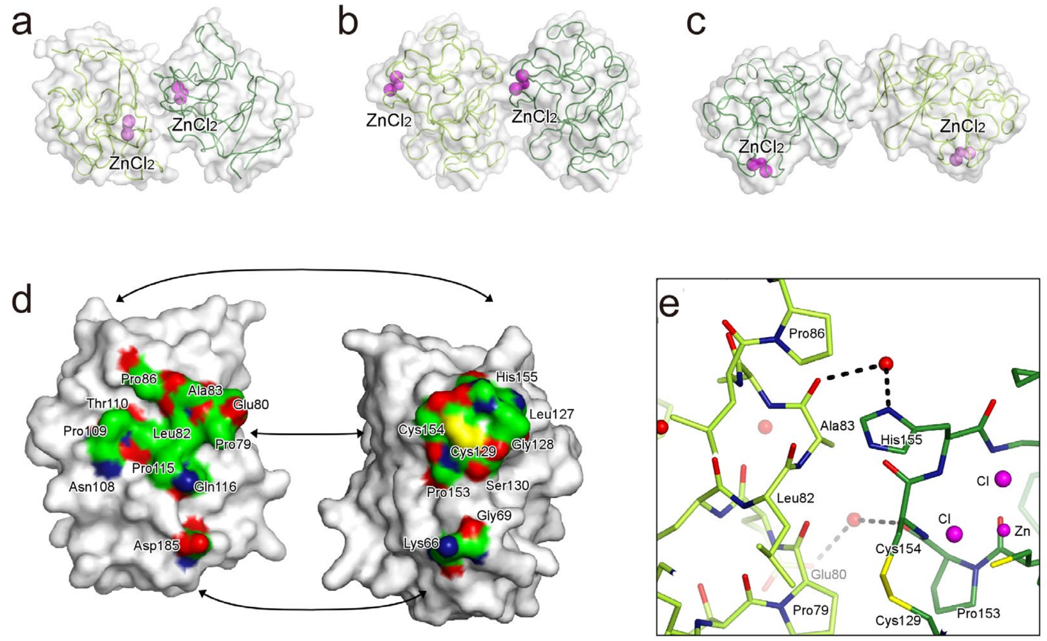

Supplementary Figure 9 : Dimer structures constructed from the crystal packing. (**a**) A dimer structure (Model I) in which two protomers are related by a crystallographic 2_1 axis. One protomer is colored in green, while another protomer is colored in yellow-green. $ZnCl_2$ is shown as magenta spheres. A semi-transparent white surface of the dimer is overlaid. **(b)** A dimer structure (Model II) in which two protomers are related by a translational symmetry. **(c)** A dimer structure (Model III) in which two protomers are related by a non-crystallographic 2-fold axis. (**d**) The interface of the inter-protomer interaction in Model II. The surfaces of the interface atoms are colored by atom type (carbon: green; nitrogen: blue; oxygen: red; sulfur: yellow). **(e)** A close-up view of the dimer interface in Model II. Hydrogen bondings via water molecules are indicated by black dotted lines.

Supplementary Table 1 : Conditions for the data collection and crystallographic statistics

	Zn-TtPetA		[2Fe-2S]-TtPetA		
**Data collection**					
Wavelength (Å)	1.28	1.29	1.00	1.74	1.75
Temperature (K)	15	15	15	15	15
**Crystal data**					
Space group	$P2_1$	$P2_1$	$P2_1$	$P2_1$	$P2_1$
Cell parameters a (Å)	32.60	32.62	32.41	32.56	32.61
b (Å)	98.71	98.75	51.02	53.13	53.28
c (Å)	51.05	51.07	98.81	96.43	96.52
β (°)	90.03	90.01	93.45	93.25	93.24
Resolution range (Å)	50–1.70 (1.80–1.70)	50–1.70 (1.80–1.70)	50–1.79 (1.90–1.79)	50–2.79 (2.96–2.79)	50–2.79 (2.96–2.79)
Total reflections	241,958	242,220	103,445	55,146	55,198
Unique reflections	34,104	34,156	30,162	8,316	8,340
Completeness (%)	95.7 (92.5)	95.6 (92.4)	99.4 (98.4)	99.2 (95.6)	99.4 (96.9)
$I/\sigma(I)$	15.9 (4.7)	17.8 (4.9)	10.1 (3.2)	9.8 (4.0)	11.3 (4.6)
R_{sym}[a] (%)	7.8 (30.3)	6.9 (33.1)	8.4 (32.3)	16.8 (39.0)	14.5 (34.9)
R_{meas}[b] (%)	8.4 (32.7)	7.5 (33.1)	9.9 (38.4)	18.2 (42.5)	15.7 (38.1)
$CC_{1/2}$ (%)	99.9 (96.4)	99.9 (96.3)	99.5 (92.7)	99.4 (96.8)	99.5 (97.3)

Values for the highest resolution shell are in parentheses.

[a] $R_{sym} = \Sigma_{hkl}\Sigma_i | I_{hkl,I} - \langle I_{hkl}\rangle | / \Sigma_{hkl}\Sigma_i I_{hkl,i}$.

[b] $R_{meas} = \Sigma_{hkl}(\sqrt{n_{hkl}/(n_{hkl}-1)})\Sigma_i | I_{hkl,I} - \langle I_{hkl}\rangle | / \Sigma_{hkl}\Sigma_i I_{hkl,i}$.

Supplementary Table 2 : *B*-factors of atoms in the cluster binding site.

Form	Atom	*B*-factor in chain A (Å^2)	*B*-factor in chain B (Å^2)
Zn-TtPetA	Zn	14.67	14.00
(7YR9)	Cl1	14.27	14.17
	Cl2	17.48	18.41
	Cys124 Sγ	15.09	17.03
	His126 Nδ1	17.08	15.96
	Cys152 Sγ	14.06	14.19
	His155 Nδ1	23.04	21.66
[2Fe-2S]-TtPetA	Fe1	16.15	16.51
(7YRA)	Fe2	17.41	17.12
	S1	16.46	16.64
	S2	15.69	15.98
	Cys124 Sγ	16.97	15.10
	His126 Nδ1	20.49	20.31
	Cys152 Sγ	15.94	18.06
	His155 Nδ1	18.90	20.02

Occupancies for all atoms were fixed to be 1.00 in the refinement calculations.

His126 Nδ1 and His155 Nδ1 have no direct interactions with Zn, Cl1 and Cl2 in Zn-TtPetA.

Supplementary Table 3 : Conditions for the data collection and crystallographic statistics.

	Br-soak	
**Data collection**		
Wavelength (Å)	0.91	0.93
Temperature (K)	100	100
**Crystal data**		
Space group	$P4_3$	$P4_3$
Cell parameters *a* (Å)	58.55	58.35
b (Å)	58.55	58.35
c (Å)	50.41	50.30
Resolution range (Å)	50–1.79 (1.90–1.79)	50–1.80 (1.91–1.80)
Total reflections	218,276	218,485
Unique reflections	16,086	15,879
Completeness (%)	99.7 (98.5)	99.9(99.5)
$I/\sigma(I)$	14.2 (2.0)	27.9(7.1)
R_{sym}[a] (%)	14.6 (122.9)	6.8(33.2)
R_{meas}[b] (%)	15.2 (127.8)	7.1 (34.4)
$CC_{1/2}$ (%)	99.9 (82.0)	100.0(98.6)

Values for the highest resolution shell are in parentheses.

[a] $R_{sym} = \Sigma_{hkl}\Sigma_i | I_{hkl,i} - \langle I_{hkl}\rangle | / \Sigma_{hkl}\Sigma_i I_{hkl,i}$.

[b] $R_{meas} = \Sigma_{hkl}(\sqrt{n_{hkl}/(n_{hkl}-1)})\Sigma_i | I_{hkl,i} - \langle I_{hkl}\rangle | / \Sigma_{hkl}\Sigma_i I_{hkl,i}$.

Supplementary References

1. Larkin, M. A., Blackshields, G., Brown, N. P., Chenna, R., McGettigan, P. A., McWilliam, H., Valentin, F., Wallace, I. M., Wilm, A., Lopez, R., Thompson, J. D., Gibson, T. J. & Higgins, D. G. Clustal W and Clustal X version 2.0. *Bioinformatics* **23**, 2947−2948 (2007).

2. Holm, L., Laiho, A., Toronen, P. & Salgado, M. DALI shines a light on remote homologs: one hundred discoveries. *Protein Sci.* **23**, e4519 (2023).

3. Kolling, D. J., Brunzelle, J. S., Lhee, S., Crofts, A. R. & Nair, S. K. Atomic resolution structures of Rieske iron-sulfur protein: role of hydrogen bonds in tuning the redox potential of iron-sulfur clusters. *Structure* **15**, 29−38 (2007).

4. Kleinschroth, T., Castellani, M., Trinh, C. H., Morgner, N., Brutschy, B., Ludwig, B. & Hunte, C. X-ray structure of the dimeric cytochrome bc_1 complex from the soil bacterium *Paracoccus denitrificans* at 2.7-Å resolution. *Biochim. Biophys. Acta.* **1807**, 1606−1615 (2011).

5. Solmaz, S. R. & Hunte, C. Structure of complex III with bound cytochrome *c* in reduced state and definition of a minimal core interface for electron transfer. *J. Biol. Chem.* **283**, 17542−17549 (2008).

6. Iwata, S., Saynovits, M., Link, T. A. & Michel, H. Structure of a water soluble fragment of the 'Rieske' iron-sulfur protein of the bovine heart mitochondrial cytochrome bc_1 complex determined by MAD phasing at 1.5 Å resolution. *Structure* **4**, 567−579 (1996).
