# Supplementary material for: Structure of a putative immature form of a Rieske-type iron-sulfur protein in complex with zinc chloride
Source: Commun Chem. 2023 Sep 9;6:190. doi: 10.1038/s42004-023-01000-6 (PMC10492824; doi:10.1038/s42004-023-01000-6)
Supplement: Supplementary file 3 — Description of Additional Supplementary Files [file 42004_2023_1000_MOESM3_ESM.pdf]

Description of Additional Supplementary Files

File name: Supplementary Data 1

Description: Structure of Zn-TtPetA (PDB ID: 7YR9)

File name: Supplementary Data 2

Description: Structure of [2Fe-2S]-TtPetA (PDB ID: 7YRA)
